# Recent Developments in Non-Conventional Welding of Materials

**DOI:** 10.3390/ma15010171

**Published:** 2021-12-27

**Authors:** Rui M. Leal, Ivan Galvão

**Affiliations:** 1 LIDA-ESAD.CR, Instituto Politécnico de Leiria, Rua Isidoro Inácio Alves de Carvalho, 2500-321 Caldas da Rainha, Portugal; rui.leal@ipleiria.pt; 2 CEMMPRE, Departamento de Engenharia Mecânica, University Coimbra, Rua Luís Reis Santos, 3030-788 Coimbra, Portugal; 3 ISEL, Departamento de Engenharia Mecânica, Instituto Politécnico de Lisboa, Rua Conselheiro Emídio Navarro 1, 1959-007 Lisbon, Portugal

## 1. Introduction and Scope

Welding is one of the technological fields with the greatest impact in many industries, such as automotive, aerospace, energy production, electronics, the health sector, etc. Welding technologies are currently used to join the most diverse materials, from metallic alloys to polymers, composites, or even biological tissues. Despite the relevance and wide application of traditional welding technologies, these processes do not meet the demanding requirements of some industries. This has driven strong research efforts in non-conventional welding processes, such as laser welding, ultrasonic welding, impact welding, friction stir welding (FSW), diffusion welding, and many other welding technologies. Important studies have been recently developed all over the world on the application of these processes to the joining of cutting-edge materials and material combinations, enabling the production of joints with improved properties. Thus, this Special Issue presents a sample of the most recent developments in the non-conventional welding of materials, which will drive the design of future industrial solutions with increased efficiency and sustainability.

## 2. Contributions

The present Special Issue encompasses the publication of eight research papers and three review papers. Literature analysis, experimental and experimental/numerical research approaches were followed in the published papers, which were focused on several welding technologies (FSW; explosive welding; laser welding, etc.) and on varied materials and material combinations (steels; aluminium, copper, and high entropy alloys; aluminium/titanium/magnesium and zirconium/titanium/steel combinations, etc.). Profound multi-scale characterisations, focused on a large range of engineering topics, such as weld macro- and microstructures, mechanical behaviour, chemical and phase composition, and supported literature-based discussions are presented in these works. 

FSW was the most addressed welding process in the works published in this Special Issue. Khan et al. [1] studied the effect of the strain rate and the heat generation on the traverse force registered during dissimilar FSW of the 2219 and 7475 aluminium alloys. These authors estimated the strain rate by grain size and welding temperature analysis, and they found that maximum values of traverse force are associated with higher strain rates. Additionally, the authors found that the strain rate mostly depends on the tool traverse speed than on the tool rotation speed. However, according to them, the effect of the rotation speed on the flow stress, which manifests by the frictional heating, is dominant when compared to the strain rate strengthening. In the same year, Tamadon et al. [2] conducted a work focused on characterising the microstructure of the flow patterns in bobbin FSW of the 6082 aluminium alloy. The authors reported that one of the main difficulties in bobbin FSW is the visualisation of the weld microstructure, especially when materials with a fine grain structure are welded. Thus, they were able to characterise the microstructure of the flow lines observed in the weld macrostructure by combining optical microscopy and electron backscatter diffraction (EBSD). These structures were found to be composed of fine layers of materials of about 10 µm thickness, which were formed by dynamic recrystallisation.

Unlike the previous works, in which butt joining was addressed, Manuel et al. [3] studied the influence of the base material properties and the traverse speed on the morphology and mechanical behaviour of tri-dissimilar AA2017/AA6082/AA5083 T-joints produced by FSW. These authors reported that the traverse speed and the base material properties and relative position in the joint have a strong influence on nugget formation. According to them, improved static and fatigue behaviour of the welds can be achieved by increasing the traverse speed. Moreover, they found that higher peak temperatures and better fatigue properties are achieved when the 2017 aluminium alloy is located at the advancing side of the weld. 

The application of the FSW process to other materials beyond the aluminium alloys and to dissimilar material combinations was also addressed in the present Special Issue. Machniewicz et al. [4] studied the effect of the traverse speed on the mechanical properties of copper FS welds. For the traverse speed range tested by the authors, the effect of this parameter on the static and especially on the fatigue behaviour of the welds was found to be stronger than its effect on the weld macro and microstructure. Considering the base material and the tested rotation speed and tool design, the authors reported that intermediate values of the traverse speed range provide welds with better mechanical properties. They still stressed that, among all the inspections performed in this work, fatigue testing was revealed as the most effective procedure for assessing the weld quality. Regarding the FSW of dissimilar materials, Chitturi et al. [5] studied the influence of the tilt angle and the pin depth on the structural and mechanical properties of AA5052/304 stainless steel joints. Following a design of experiments based on Taguchi’s orthogonal array, a very strong influence of the tilt angle on the weld morphology was noticed. The authors reported that the increase in the tilt angle prevented the formation of defects and promoted the generation of intermetallic compounds (IMCs). According to them, the formation of IMCs was also influenced by the pin depth, increasing with increments in this parameter. The authors also observed that the maximum tensile strength was achieved for higher values of pin depth and tilt angle.

Besides the original research papers, one review work on FSW was also published in this Special Issue. This work, which was conducted by Laska and Szkodo [6], was focused on presenting an overview of the influence of the welding parameters on the microstructural and mechanical properties of similar and dissimilar butt welds in a large range of materials, i.e., aluminium, magnesium, steel and ferrous alloys, titanium, copper, polymers, and composites. The authors highlighted the influence of the tool traverse and rotational speeds, tool geometry and material, tilt angle, tool offset, plunge depth, and axial force on the welding conditions. For dissimilar welding, they stressed the recent trends of using interlayers or water environments to minimise the formation of detrimental IMCs. The authors also recommended more understanding of the material flow and further work on the characterisation of the weld electrochemical properties. 

FSW was not the only solid-state process addressed in the present Special Issue. Two of the published works were on explosive welding. One of these works, which was developed by Wachowski et al. [7], was aimed at studying the effect of the post-welding hot-rolling on the structural and mechanical properties of magnesium/aluminium/titanium composites. The authors reported that the composites were affected by the strain and temperature experienced during hot-rolling. However, they referred that important differences in structure and mechanical behaviour existed for both weld interfaces. While a clear trend in the evolution of the interfacial strength and the rolling temperature was registered for the magnesium–aluminium interface, a less clear trend was registered for the other interface. In terms of microstructure, the hot-rolling promoted the formation of continuous IMC layers at the magnesium/aluminium interface and the precipitation of IMCs in the molten regions of the aluminium/titanium interface. In the other work on explosive welding, Karolczuk et al. [8] studied the influence of the impact velocity on the residual stresses, strength, and structural properties of zirconium/titanium/steel composites. According to the authors, the tested differences in impact velocity did not promote strong changes in the microhardness distribution and in the tensile yield force. Regarding the residual stresses, they found that the compressive stresses initially present in the as-received zirconium alloy decreased in the welding process, resulting in tensile stresses. Additionally, the authors observed that a higher impact velocity led to higher tensile stresses in the zirconium, and therefore, a lower impact velocity is recommended to protect the composite from stress-based corrosion cracking. 

A paper on laser welding was published by Danielewski et al. [9], who studied dissimilar lap welding of the S355J2 and 316L steels under the two alternative positions of the base material plates. The authors, following an experimental/numerical approach, concluded that the relative position of the base materials has a strong effect on the welding results. According to them, better weld properties are obtained by placing the stainless steel (316L) on the top of the joint. The authors found that this position of the plates promoted a maximum principal stress value, lower differences in the distribution of the chemical elements over the weld, and a more uniform weld structure and fusion rate.

Review works addressing the welding issues of two specific material classes were also published in the present Special Issue. The study conducted by Parker and Siefert [10] was developed based on the Electric Power Research Institute’s works on the manufacture and performance of welds in creep strength-enhanced ferritic steels. The authors refer that these works have enabled identifying and quantifying the factors affecting the high-temperature performance of these steels. According to them, the resulting knowledge has been used to support recommendations for improving the production and control of components in creep strength-enhanced ferritic steels. In turn, Filho and Monteiro [11] addressed the recent trends in the welding of high entropy alloys. The authors found that both fusion welding and solid-state welding techniques could be used to join these materials. They also stressed that the works already developed in this field have focused on varying aspects, such as grain refinement, dynamic recrystallization, hardness enhancement, secondary phase precipitation, ductility decrease, and strengthening of the material. Furthermore, the authors reported that, for welding techniques with high power density, the loss of elements with low melting and evaporation temperatures was a challenge for controlling the chemical composition of the welded alloys.

## 3. Conclusions and Outlook

A sample of the cutting-edge research currently conducted in non-conventional welding of materials was presented in this Special Issue. The demanding methodological approaches followed in the different works, coupled with the supported discussions, allowed solid conclusions to be achieved, which strongly contributed to enriching the knowledge in this field. Even so, future research in this area keeps pertinent and relevant so that engineering solutions meeting the increasingly demanding industrial criteria can be developed. 

As the Editors of the present Special Issue, we consider that it was a very successful project. This would be impossible without the precious contribution of all the authors and the reviewers. The quality, rigour, scientific relevance, and actuality of the papers submitted by the authors, which were ensured and potentiated by supported and rigorous reviews, are the main strength of this project. We are profoundly grateful to all the authors and the reviewers. Finally, we acknowledge all support provided by the Materials editorial team, especially by Elsa Qiu, during the development of this Special Issue.

## References

[1] Khan N.Z., Bajaj D., Siddiquee A.N., Khan Z.A., Abidi M.H., Umer U., Alkhalefah H. (2019). Investigation on Effect of Strain Rate and Heat Generation on Traverse Force in FSW of Dissimilar Aerospace Grade Aluminium Alloys. Materials.

[2] Tamadon A., Pons D.J., Clucas D., Sued K. (2019). Texture Evolution in AA6082-T6 BFSW Welds: Optical Microscopy and EBSD Characterisation. Materials.

[3] Manuel N., Galvão I., Leal R.M., Costa J.D., Loureiro A. (2020). Nugget Formation and Mechanical Behaviour of Friction Stir Welds of Three Dissimilar Aluminum Alloys. Materials.

[4] Machniewicz T., Nosal P., Korbel A., Hebda M. (2020). Effect of FSW Traverse Speed on Mechanical Properties of Copper Plate Joints. Materials.

[5] Chitturi V., Pedapati S.R., Awang M. (2019). Effect of Tilt Angle and Pin Depth on Dissimilar Friction Stir Lap Welded Joints of Aluminum and Steel Alloys. Materials.

[6] Laska A., Szkodo M. (2020). Manufacturing Parameters, Materials, and Welds Properties of Butt Friction Stir Welded Joints–Overview. Materials.

[7] Wachowski M., Kosturek R., Śnieżek L., Mróz S., Stefanik A., Szota P. (2020). The Effect of Post-Weld Hot-Rolling on the Properties of Explosively Welded Mg/Al/Ti Multilayer Composite. Materials.

[8] Karolczuk A., Kluger K., Derda S., Prażmowski M., Paul H. (2020). Influence of Impact Velocity on the Residual Stress, Tensile Strength, and Structural Properties of an Explosively Welded Composite Plate. Materials.

[9] Danielewski H., Skrzypczyk A., Hebda M., Tofil S., Witkowski G., Długosz P., Nigrovič R. (2020). Numerical and Metallurgical Analysis of Laser Welded, Sealed Lap Joints of S355J2 and 316L Steels under Different Configurations. Materials.

[10] Parker J., Siefert J. (2019). Manufacture and Performance of Welds in Creep Strength Enhanced Ferritic Steels. Materials.

[11] Garcia Filho F.C., Monteiro S.N. (2020). Welding Joints in High Entropy Alloys: A Short-Review on Recent Trends. Materials.

